# Heat Shock Proteins: Protection and Potential Biomarkers for Ischemic Injury of Cardiomyocytes After Surgery

**DOI:** 10.21470/1678-9741-2017-0169

**Published:** 2018

**Authors:** Valfredo de Almeida Santos-Junior, Pablo Christiano Barboza Lollo, Marcos Antonio Cantero, Carolina Soares Moura, Jaime Amaya-Farfan, Priscila Neder Morato

**Affiliations:** 1 Faculdade de Engenharia de Alimentos (FEA) da Universidade Estadual de Campinas (Unicamp), Campinas, SP, Brazil.; 2 Faculdade de Ciências da Saúde (FCS) da Universidade Federal da Grande Dourados (UFGD), Dourados, MS, Brazil.

**Keywords:** Heat-shock response, Cytoprotection, Biomarkers, Myocardium, Infarction

## Abstract

The heat shock proteins are endogenous proteins with the ability to act as molecular chaperones. Methods that provide cell protection by way of some damage can positively influence the results of surgery. The present review summarizes current knowledge concerning the cardioprotective role of the heat shock proteins as occurs in heart damage, including relevant information about the stresses that regulate the expression of these proteins and their potential role as biomarkers of heart disease.

**Table:** 

Abbreviations, acronyms & symbols	
GGC	= Geranylgeranylacetone
HSPs	= Heat shock proteins

## INTRODUCTION

Heat shock proteins (HSPs) are a family of endogenous proteins responsible for a variety of stresses. The are classified according to their molecular weights in families, *e.g*. HSP27, HSP70, etc.^[^^1^^]^. They have the ability to act as 'molecular chaperones', since they stabilize macromolecules, guide protein folding, perform the refolding and remove irreversibly denatured proteins in the cell^[^^2^^-^^4^^]^.

The HSPs can be overexpressed in various stress situations, such as hyperthermia^[^^5^^,^^6^^]^, hemodynamic stress caused by heart diseases^[^^7^^]^, physical exercise^[^^8^^]^, the administration of some substances as geranylgeranylacetone^[^^9^^]^ and glutamine^[^^10^^]^, among others.

Heart surgery improves the survival and clinical prognosis of various diseases, but can induce an ischemic/reperfusion condition that damages the cardiac tissue. Methods that induce heart protection by ischemic damage can positively influence the result of surgery. Some HSPs have been the target of studies because they increase the resistance of myocardium cells against ischemia^[^^11^^-^^14^^]^. Other studies verified the relationship between HSPs and the development of heart disease^[^^15^^-^^17^^]^. Since failures in the detection of heart diseases can worsen the odds, more sensitive methods could significantly increase patient survival.

The expression of HSPs in the heart has been the focus of several studies, but some questions still need clarification, such as: do all HSPs have a protective effect? Would increases in HSPs serve as new biomarkers for the diagnosis/prognosis of cardiovascular diseases? Would the increase offer advantages/improvements in the clinical outcome of cardiovascular diseases? As from these doubts, the objective of the present study was to systematically analyze the published studies concerning the expression of HSPs in the heart.

## METHODS

**Fig. 1:**
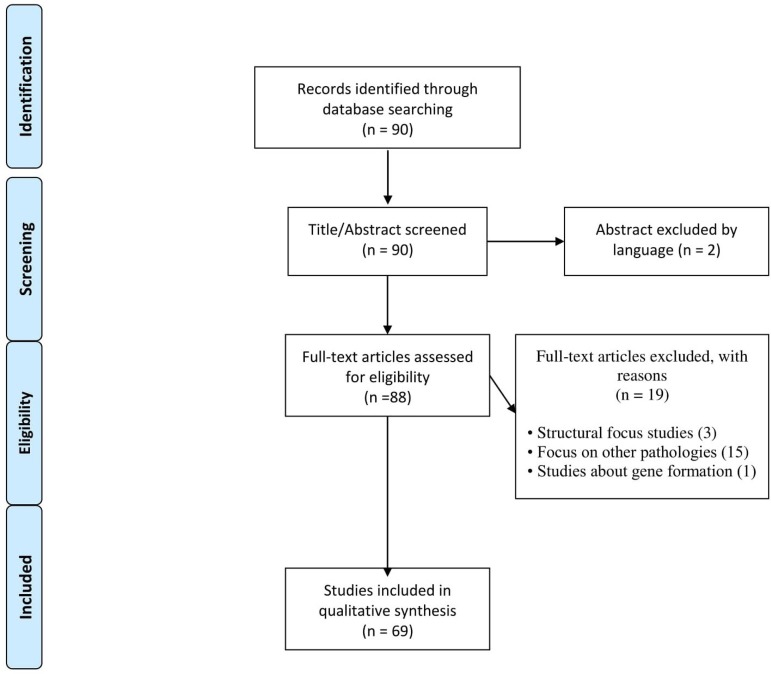
Flow diagram showing the inclusion and exclusion criteria of the articles.

The literature survey was carried out based on the PubMed data using the descriptors "Heat shock protein" and "heart" as components of the search field title. The objective was to select articles that researched the expression of HSPs specifically in the heart. We found 90 articles, among which those that included the objectives of the search were selected, excluding articles in languages other than English, and texts that were not complete articles or made conclusions about other substances/means or that did not report directly on the problems/cardiac tissues (Figure 1).

## RESULTS AND DISCUSSION

### HSPs and Heart Protection

HSP70 and some other small heat shock proteins (sHSPs) were found to provide heart protection. Of the 69 articles included in this study, 26 dealt with the effect of the HSPs in heart protection and 25 showed that the presence of these proteins was associated with the protective effect in cardiac tissue^[^^5^^,^^9^^,^^11^^-^^14^^,^^18^^-^^36^^]^.

Part of the myocardial protection granted, according to the authors, was due to the effect of HSP70 in response to ischemic damage. Yamashita et al.^[^^18^^]^ and Kukreja et al.^[^^19^^]^ induced the superexpression of HSP70, thus obtaining a significant reduction in the infarcted area. Qian et al.^[^^20^^]^, Okubo et al.^[^^12^^]^, Vittorini et al.^[^^14^^]^, Zhao et al.^[^^32^^]^ and Li et al.^[^^34^^]^ reported similar results. Additionally, Yamashita et al.^[^^18^^]^ showed that HSP70 content correlated with the time course of cardioprotection. Furthermore, an increase in HSP70 expression can prevent lipopolysaccharide-induced dysfunction^[^^21^^]^. Only Xi et al.^[^^37^^]^ did not observe diferences in infarct size between the HSP70 and control groups.

In addition to the decrease in the infarcted area, an increase in the expression of HSP70 offers an improvement in the recovery of post-ischemia/reperfusion injury^[^^9^^,^^11^^,^^22^^,^^24^^,^^28^^]^. Nomura et al.^[^^22^^]^ verified that upregulation of HSP70 before cardioplegic ischemia improved the recovery of systolic and coronary endothelian function. Ooie et al.^[^^24^^]^ demonstrated that an increase in HSP70 expression induced by geranylgeranylacetone (GGC) significantly improved post-ischemia heart recovery and decreased the cardiac injury markers.

Tanonaka et al.^[^^5^^]^ demonstrated that an increase in HSP70 was inversely correlated with worsening of cardiac parameters. However, an infarcted heart appears to have a lower production capacity of HSP70, which could be intimately related to its functional deterioration and ability to tolerate further damage^[^^5^^]^. An increase of HSP70 expression is also correlated with a decrease in heart apoptosis^[^^26^^,^^30^^]^. Both authors explored the expression of this protein as related to changes in endogenous hormones, but the role of these hormones on the expression of HSP70 is still unclear.

In addition, sHSPs also promote heart protection^[^^13^^,^^23^^,^^25^^,^^27^^,^^29^^,^^31^^,^^33^^,^^35^^]^. Kim et al.^[^^23^^]^, Efthymiou et al.^[^^25^^]^ and Kwon et al.^[^^13^^]^ found that HSP27 offered a protective effect in cases of infarction. Groups that overexpressed HSP27 presented significant reductions in the infarcted areas and reductions in cell apoptosis in cardiac tissue. Zhu and Wang^[^^29^^]^ observed these same characteristics with increased expression of HSP20.

Chen et al.^[^^27^^]^ reveals that type-1 diabetic hearts are resistant to ischemic injury by upregulation of phosphorylated HSP27 and the low expression of HSP27 was associated with atrial fibrillation in patients with rheumatic heart disease^[^^36^^]^. An increase in the expression of HSP27 also provided an increase in the efficiency of stem cell therapy in the myocardial recovery, decreasing cell apoptosis and improving heart recovery during therapy^[^^35^^]^.

Jiang et al.^[^^33^^]^ obtained similar results with increased expression of HSP32, which promoted heart protection following ischemia/reperfusion. An increase in the expression of HSP25 improved survival in patients with cardiomyopathy and increased heart resistance against toxicity^[^^31^^]^.

### Induction of the Expression of HSPs

**Fig. 2:**
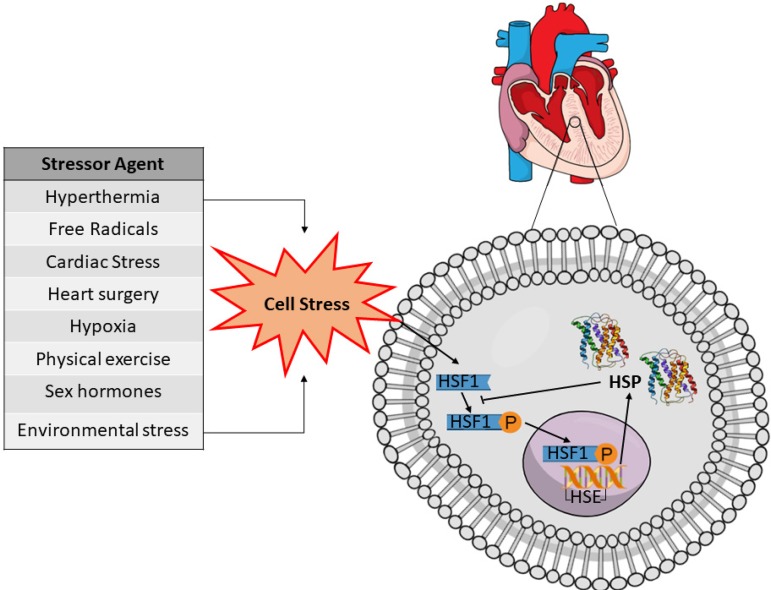
General scheme of some of the physiological signals that activate the inducible form of the heat shock protein (HSP) in the cardiac cell. Physiological stress is required to enable access to heat shock factor-1 (HSF-1) complex present in the cytosol, allowing its phosphorylation (P) by protein kinases to their active form. These HSF-1 phosphorylated complexes enter the nucleus and bind to heat shock elements (HSE) in the promoter region of the HSP-especific gene. Transcriptional and translational processes increase HSP expression in the cellular cytosol.

Hyperthermia is one of the main and best known inducers of HSP expression, and of the articles included in this systematic review, twelve used this method to increase the protein expression^[^^5^^-^^7^^,^^18^^-^^20^^,^^22^^,^^26^^,^^30^^,^^37^^-^^39^^]^. However, depending on tissue type and HSP, increased expression can be influenced by several other types of stress, as shown in the general scheme of Figure 2.

### HSP70

Some substances also influence the regulation of HSP70: circulating hormones like phenyleprine and vasopressin^[^^38^^]^, free radicals^[^^40^^]^, treatment with geranylgeranylcetone^[^^9^^,^^24^^]^, liposomal protein delivery of HSP70^[^^21^^]^, intravenous injection of anandamide^[^^34^^]^, chronic administration of *Terminalia arjuna*^[^^28^^]^, injection of HSP70 adenovirus^[^^12^^]^, probiotic-derived proteins^[^^32^^]^ and parenteral administration of glutamine^[^^10^^]^. All these were shown to be effective in increasing the expression of this protein.

Pathologies induce systematic stress that could superexpress HSP70 in the heart. Wei et al.^[^^15^^]^ verified that the protein expression of HSP70 is frequently increased in hearts showing failure due to arrhythmogenic cardiomyopathy, dilated cardiomyopathy and ischemia. Ferrari et al.^[^^41^^]^ verified that congestive heart failure increases HSP72 expression more pronounced in right than left ventricles, whereas hibernation increases expression in both. The initial stages of heart failure^[^^42^^,^^43^^]^, elevation of aortic pressure^[^^44^^]^ and diabetes mellitus^[^^10^^]^ increase the expression of HSP70 in the heart tissue as a form of protection.

However, as failure proceeds, repression of the nuclear portion of HSF-1 (heat shock factor 1) ensues thus inhibiting the expression of HSP70 in the more serious stages of the disease^[^^5^^,^^7^^,^^43^^-^^46^^]^. Two studies verified the involvement of diabetes mellitus in HSP70 expression^[^^10^^,^^27^^]^. Ugurlucan et al.^[^^10^^]^ demonstrated an increase of HSP70 in diabetic hearts, while Chen et al.^[^^27^^]^ observed no differences between the control and diabetic groups. Further research is essential to clarify the effects of diabetes mellitus on the expression of HSP70 in the heart.

Steroid hormones alter the expression of HSP70 differently in men and women. Treatment with 17-B-estradiol or progesterone can activate HSF-1 and consequently increase the HSP70 expression, but not of the other HSPs^[^^47^^]^. Shinohara et al.^[^^26^^]^ found that male hearts are more sensitive to the induction of HSP70 and the author explained that this finding was due to the inhibitory effect of estrogen on the HSP70 expression. However, Kohno et al.^[^^30^^]^ showed that testosterone also had an inhibitory effect on the expression of HSP70, this inhibition being mediated by testosterone receptors in the heart tissue. Further research is required in this field, since the analysis of the results of these two studies seems to indicate that both sex hormones have an inhibitory effect, although estrogen may be a more potent inhibitor than testosterone, unless other factors are involved in the regulation process.

The stress produced by the heart surgery itself has been shown to induce an increase in HSP70 expression. Schmitt et al.^[^^48^^]^ reported a superexpression of HSP70 after stress caused by cardioplegic arrest, which was proportional to the duration of the cardioplegia. The increase became more pronounced after two hours, leading to the conclusion that the synthesis peaks at about two hours in human hearts. There were no significant changes in the HSPs of other molecular weights.

Similar results were found by Vittorini et al.^[^^14^^]^ and Dybdahl et al.^[^^49^^]^, who observed that cardioplegia positively regulated the expression of HSP70. Ischemia/reperfusion preconditioning upregulates the HSP70 expression^[^^20^^,^^50^^]^. Hypothermic cardioplegia showed increase the HSP70 expression even more than normothermic controls, but only one study tested this hypothesis and this topic needs more research^[^^51^^]^.

Other types of cardiac stress have been shown to be efficient in increasing HSP70 expression, such as height-induced hypoxia, remaining high for up to two weeks^[^^11^^]^ or pulmonar artery banding^[^^52^^]^, a single stretch and fiber shortening^[^^53^^]^, physical exercise^[^^8^^]^ and stress caused by environmental changes^[^^54^^]^.

### HSP60

The increase in HSP60 expression due to hyperthermia is tissue-specific. Yan et al.^[^^55^^]^, verified the behavior of HSP60 under acute heat conditions and showed that the expression was tissue-specific and that the increase was related to the extent of damage to the tissue. In the heart, HSP60 started increasing after one hour of induction and reached a peak after five hours.

On analyzing the protein expression induced by the development of heart failure, Tanonaka et al.^[^^43^^]^ showed that HSP60 levels only increased in the eighth week, when functional changes occurred that defined the presence of the pathology. Hoppichler et al.^[^^56^^]^ found an increase in HSP60 antibodies in chronic heart disease. Wang et al.^[^^46^^]^ demonstrated that the increase in HSP60 during heart failure could be mediated by the increase in circulating NFkB. In the reviewed articles, only three dealt with forms of inducing HSP60 expression.

### Small Heat Shock Protein (HSP20, 25 and 27)

Stress induced by some diseases affects the expression of HSP27 and some other HSPs. Tanonaka et al.^[^^43^^]^ found that at the onset of heart failure there was an increase in HSP27. Corroborating the finding above, Dohke et al.^[^^57^^]^ observed an increase in phosphorylation of HSP20 and HSP27. Ischemic preconditioning also increases HSP27 expression^[^^23^^]^. Hu et al.^[^^58^^]^ demonstrated a reduction in the expressions of HSP27 and HSP32 in the heart following intracerebral hemorrhage, but treatment with deferoxamine reversed the reduction in HSP32, although making the reduction in HSP27 even more pronounced.

Raju et al.^[^^45^^]^ observed that congestive heart failure increases HSP32 without changing HSP70 expression, showing that HSPs behave in distinct manners with each other. The regulation of some sHSP may be correlated with endogenous proteins. As published by Jiang et al.^[^^33^^]^, nucleolin interacts with the mRNA of HSP32 increasing its stability and consequently its expression.

McGinley et al.^[^^35^^]^ noted that it was possible to increase the expression of HSP27 by treating with lentivirus vectors, an effect similar to that demonstrated by Kwon et al.^[^^13^^]^, which induced an increase in HSP27 using a protein delivery system by recombinant HSP27 linked to a protein transduction domain. The adenoviruses HSP20 and HSP22 were also efficient in increasing the expression of these proteins in cardiomyocytes^[^^29^^]^. Pretreatment with HSP25 enriched plasma also induced an increase in the expression of extracellular HSP25, according to a study published by Krishnamurthy et al.^[^^31^^]^.

Systemic stress positively regulate the expression of HSPs. Physical exercise increased the expression of HSP27^[^^8^^]^, and Boluyt et al.^[^^59^^]^ showed that only chronic physical training caused an increase in HSP20 expression, which persisted for at least 72 hours of detraining. Stresses such as drug abstinence induced an increase in HSP, as verified by the work of Almela et al.^[^^60^^]^, where morphine-dependent rats showed an increase in HSP27 expression upon receiving saline instead of morphine.

### HSPs as Potential Biomarkers for Heart Disease

Due to their characteristic response to diverse stresses, including heart disease, the power of HSPs as diagnostic and prognostic markers for heart disease has been investigated. Of the papers included in this study, 21 of them verified this relationship^[^^5^^,^^15^^,^^17^^,^^42^^,^^45^^,^^49^^,^^56^^,^^57^^,^^61^^-^^72^^]^.

Of these studies, nine investigated the relationship of HSP60 expression and its potential to detect heart disease, and seven of them showed that HSP60 had the potential to be a diagnostic or prognostic marker of heart disease. Veres et al.^[^^64^^]^ and Zhang et al.^[^^70^^]^ verified that high levels of HSP60 could increase the risk of heart disease and could be considered as a new familial risk factor for these diseases.

Elevated HSP60 concentrations were positively associated with the severity of coronary arterial disease in a dose-dependent way^[^^70^^,^^71^^]^ and with ischemic heart disease^[^^68^^]^, and also showed a correlation with heart failure and other adverse cardiac events and antibodies levels in sera can be correlated with worse prognosis^[^^16^^,^^61^^,^^69^^]^. Only Hoppichler et al.^[^^56^^]^ and Rothenbacher et al.^[^^63^^]^ reported that high levels of HSP60 did not correlate with the risk factor for heart disease.

Of the ten studies that verified the role of HSP70 as a possible biomarker for heart disease, nine came to a positive conclusion. The pioneering work of Comini et al.^[^^42^^]^ was confirmed by Genth-Zotz et al.^[^^65^^]^ and Gombos et al.^[^^67^^]^, who found that the levels of this protein were significantly higher in the groups with heart failure and that this expression was related to the severity of the disease. In agreement with those reports, Wei et al.^[^^15^^]^ also observed that an increase in the expression of HSP70 was common in heart failure.

Comini et al.^[^^42^^]^ showed that congestive heart failure, but not compensatory hypertrophy, increases HSP70 expression in heart. Only Raju et al.^[^^45^^]^ found no changes in HSP70 expression in the congestive heart failure model. Patients with myorcardial infarction also show higher levels of HSP70 than control subjects^[^^66^^]^. Another fact that supports its use as a diagnostic/prognostic biomarker of heart disease is the correlation that has been reported between HSP70 and the traditional injury markers such as AST, ALT, γGT and bilirubin in patients with heart failure^[^^5^^,^^67^^]^.

In assessing the relationship between HSP70 and progression of heart failure, Li et al.^[^^17^^]^ verified a significant increase with the progression of disease stages, showing their potential for detection, mainly in old myocardial infarction or in those whith structural heart disease. Baba et al.^[^^62^^]^ concluded that worse parameters correlate with increased HSP70 and that this increase was inversely correlated with rejection in the case of heart transplantation. Dybdahl et al.^[^^49^^]^ concluded that measurement of increased levels of HSP70 in post-cardiac surgery tissue and ischemia could offer an advantage in the diagnosis and prognosis of such cases.

Increased sHSPs are also correlated with heart failure, and HSP27 can be used as a marker for this purpose. It was shown that HSP20, HSP27 and HSP32 were involved in congestive heart failure due to significant increases in the phosphorylated forms that appear in this disease^[^^45^^,^^57^^]^. An increase in HSP27 was correlated with the progression of heart failure in animals^[^^17^^]^, and in humans HSP27 was significantly higher in patients with valvular heart disease^[^^72^^]^, suggesting its use as a marker for disease.

### Influence of HSP on Heart Health

HSPs work as a cellular defense mechanism, acting as a complementary antioxidant system; the oxidative stress inducing an increase in the expression of one or several HSPs, and this increase in turn promotes protection^[^^8^^]^ through repairing. The accumulation of reactive oxygen species throughout a lifetime, however, can affect the efficiency and homeostasis of the cellular system.

Ageing negatively affects HSP70 expression in the heart, leaving the heart more susceptible to oxidative damage, but Rinaldi et al.^[^^8^^]^ showed that physical exercise increased the expression of HSP70 and HSP27 in the heart. In fact, the expression of these proteins inhibits apoptosis and protects the integrity of actin and cardiac microtubule cytoskeleton^[^^46^^]^, thus explaining in part the beneficial effect of exercise on the heart of the elderly.

HSP70 is an endogenous activator of the innate immune system^[^^49^^,^^66^^]^. The circulating levels of HSP70 not only act as molecular chaperones but are also correlated with the decrease of inflammatory cytokines. An *in vitro* study proved the release of inflammatory cytokines mediated by HSP70, TLR4 receptor agonists^[^^66^^]^. Dybdahl et al.^[^^49^^]^ found that HSP70 did, in fact, induce an increase in IL-6 and TNF in a dose-dependent manner *via* TLR4/CD14, thus demonstrating the involvement of HSP70 in the inflammatory response.

Besides guiding the initial protein folding, some small heat shock proteins help in heart protection and function. Qiu et al.^[^^73^^]^ demonstrated that HSP22 depletion did not affect heart function under basal conditions, but following cardiac overload, its absence promoted eccentric hypertrophy and dilation of the heart, accelerated the transition to heart failure, and interfered in the activation of the cellular protection system.

Additionally, an increase in HSP25 expression can prevent apoptosis signaling, antagonizing the activation of TLR2 after systemic stress, such as in the case of toxic treatments or the accumulation of denatured proteins^[^^31^^]^. HSP20 positively interferes in the contractile capacity of the heart; the overexpression of HSP20 is a beneficial factor for heart tissue, since it acts in the cellular protection against several types of stress and simultaneously improves the contractile function^[^^59^^]^.

Table 1 presents a summary of the evidence.

**Table 1:** Summary of evidence.

Author	Objective	Results/Conclusion
Almela et al.^[^^60^^]^	To investigate the HSP27 expression during morphine dependence and withdrawal	Morphine withdrawal ↑HSP27 in heart
Baba et al.^[^^62^^]^	To determine the correlation between hemodynamic parameters and HSP70 in the early period after heart transplantation	Worsening of the parameters is correlated with ↑HSP70 / Patients who died and in case of transplant rejection was observed more ↑HSP / ↑HSP70 was inversely correlated with rejection
Boluyt et al.^[^^59^^]^	To determine the protein changes in the heart after physical training	Chronic physical training ↑HSP20 / ↑HSP20 can stay up to 72h after physical training
Bonanad et al.^[^^16^^]^	To evaluate the correlation between HSP60 and the risk of death/recurrence of acute HF	↑HSP60 related to risk of death/recurrence
Chen et al.^[^^27^^]^	To investigate the response of HSPs and tolerance of type 1 diabetic hearts to ischemia/reperfusion injury	↑HSP27 phosphorylated and ↑injury tolerance in diabetic hearts / ↔HSP70 in diabetic hearts
Comini et al.^[^^43^^]^	To investigate the HSP70 expression in lungs, liver, cardiac and skeletal muscle in congestive heart failure	CHF, but not compensatory hypertrophy ↑HSP70 in heart
Dohke et al.^[^^57^^]^	To evaluate the change of protein expression in HF	↑HSP27 and ↑HSP20 in hearts with HF
Dybdahl et al.^[^^49^^]^	To explore the release of HSP70 after CABG	↑HSP70 release after CABG / ↑HSP70 related to ↑IL-6 and ↑TNF
Efthymiou et al.^[^^25^^]^	To investigate the heart response overexpressing HSP27 to ischemia/reperfusion injury	↑HSP27 ↓the infarct size / ↑ HSP27 protected the heart from ischemia/reperfusion injury
Ferrari et al.^[^^42^^]^	To compare myocardial hibernation and congestive heart failure in HSP72 expression	Myocardial hibernation ↑HSP72 in right and left ventricles / Congestive heart failure ↑HSP72 more marked in the right than in the left ventricle
Gauthaman et al.^[^^28^^]^	To investigate the effect of *Terminalia arjuna* (TA) on HSP expression and its influence on ischemic damage	*Terminalia arjuna* treatment ↑HSP72 / Treatment ↑the recovery of cardiac function after ischemia/reperfusion
Genth-Zotz et al.^[^^65^^]^	To investigate the circulating HSP70 levels in patients with CHF	↑HSP70 in CHF patients / ↑HSP70 related to disease severity
Gombos et al.^[^^67^^]^	To investigate the clinical and biological correlation of HSP70 in HF	↑HSP70 levels were associated with disease severity in HF patients / ↑HSP70 correlated with markers of cardiac function and liver injury
Gray et al.^[^^51^^]^	Investigate the effect of hypothermic cardioplegic arrest in the expression of HSP70	The hypothermic cardioplegic ↑HSP70 more than normothermic control
Hoppichler et al.^[^^56^^]^	To investigate the association between HSP60 antibodies with coronary heart disease and acute myocardial infarction	CHD ↓HSP60 / Myocardial infarction ↓HSP60 antibodies compared to CHD
Hu et al.^[^^58^^]^	To examine the expression of HSPs in the heart after ICH	ICH ↓HSP27 and ↓HSP32 in the heart
Jafarzadeh et al.^[^^68^^]^	To evaluate HSP60 in patients with ischemic heart disease	↑HSP60 in the groups of patients compared to control groups
Jiang et al.^[^^33^^]^	To investigate the role of nucleolin in cardiac ischemia/reperfusion injury	Nucleolin ↑HSP32 / ↑HSP32 offers cardiac protection
Katayose et al.^[^^52^^]^	To examine the expression of heme oxygenase (HSP32) and HSP70 in hearts subjected to hypoxia or pulmonary artery banding	Hypoxia ↑HSP32 e ↔HSP70 in right and left ventricle / Pulmonary artery banding ↑HSP32 and ↑HSP70
Kim et al.^[^^23^^]^	To examine whether extracellular kinases are upregulated by preconditioning and whether they are required for cadioprotection	Preconditioning ↑HSP27 and ↓infarct size
Knowlton and Sun^[^^47^^]^	To investigate the relationship between HSPs and hormone receptors	17-B-estradiol or progesterone ↑HSF-1 and ↑HSP70
Knowlton et al.^[^^53^^]^	To investigate the effect of decreased systolic shortening and a single stretch on HSP70 expression	Single stretch and fiber shortening ↑HSP70
Kohno et al.^[^^30^^]^	To investigate the effects of testosterone on HSP72 expression and its relation to cardioprotection	Exogenous testosterone ↓HSP72 after heat stress / ↑ HSP72 correlates ↓cardiac apoptosis and ↑functional recovery
Krishanarmurthy et al.^[^^31^^]^	To determine the relationship between HSP25 and protection against cardiotoxicity	↑HSP25 protected the heart from cardiotoxicity
Kukreja et al.^[^^41^^]^	To examine the influence of free radicals on HSP70 expression in the heart	Free radicals ↑HSP70
Kukreja et al.^[^^19^^]^	To verify the hypothesis that inhibition of protein kinase C would block the cardioprotection mediated by heat stress	Heat stress ↑HSP70 and ↓infarct size / PKC inhibitor ↓HSP70
Kwon et al.^[^^13^^]^	To evaluate the efficacy of an intracellular delivery system in the expression of HSP27 and in cardiac protection	Intracellular delivery system ↑HSP27 / ↑HSP27 ↓apoptosis and ↓size of the infarcted area / ↑HSP27 promotes heart protection against ischemia
Latif et al.^[^^61^^]^	To quantify levels of circulating anti-HSP60 antibodies in the cardiac transplant patient	↑HSP60 antibodies in sera appear to have a worse prognosis
Li et al.^[^^34^^]^	To test the effect of anandamide in HSP72 expression and cardioprotection	Anadamide ↑HSP70 and protect the heart against ischemia/reperfusion injury
Li et al.^[^^17^^]^	To characterize the expression of circulating HSP in HF	↑HSP27 and ↑HSP70 in animals and ↑HSP70 in humans with HF / ↑HSP70 with progression of HF
Marunouchi et al.^[^^7^^]^	To analyze the HSF-1 and HSP70 kinetics after HF	↑HSP72 and ↑HSF1 in the 2^nd^ week after HF / ↓HSP72 and ↓HSF1 after heat exposure with disease progression
McGinley et al.^[^^35^^]^	To investigate the effect of exogenous HSP27 on mesenchymal stem cell therapy in the heart	↑HSP27 ↑the survival of cell therapy *in vitro* and *in vivo* / ↑HSP27 ↓apoptosis and ↑cardiac function
Meldrum et al.^[^^21^^]^	To determine the effect of liposomal delivery of HSP70 and the role in cardioprotection	Intracoronary perfusion of the liposomal protein ↑HSP70 in the myocardium / ↑HSP70 prevents LPS-induced dysfunction
Moalic et al.^[^^38^^]^	To verify the involvement of circulating hormones in HSP70 expression	Heat stress ↑HSP70 / Phenylephrine and vasopressin ↑HSP70, but angiotensin II did not
Niizeki et al.^[^^69^^]^	To examine whether HSP 60 is correlated with the severity of CHF	↑HSP60 in patients with CHF to the control group / ↑HSP60 was associated with functional classification of CHF and risk of adverse events
Nomura et al.^[^^22^^]^	To investigate the role of HSP70 in the recovery of hypothermic cardioplegic ischemia	↑HSP70 expression in 15 min after heat stress (43°) and persisted up to 24h / ↑HSP70 improved the recovery of systolic and coronary endothelial function
Okubo et al.^[^^12^^]^	To observe the effect of the overexpression of HSP70 in myocardial protection	↑HSP70 ↓myocardial infarct size / ↑HSP70 ↓the severity of ischemic injury
Ooie et al.^[^^24^^]^	To examine the role of geranylgeranyl acetone (GGA) in HSP expression in cardioprotection	Treatment ↑HSP70 dose-dependent manner with peak expression at 24 hours after administration / ↑HSP72 provides cardioprotection by ischemia/reperfusion and ↑post-ischemic recovery
Osaki et al.^[^^44^^]^	To examine the involvement of protein kinase A and protein kinase C in pressure-induced HSP70 expression	Elevation of aortic pressure ↑HSP70 / ↑HSP70 are regulated both by PKA and PKC-dependent systems
Qian et al.^[^^20^^]^	To investigate the ischemic preconditioning cardioprotection and to correlate with HSP70 expression	Heat stress and preconditioning ↑HPS70 / heat stress ↓infarct size
Qiu et al.^[^^73^^]^	To determine the function of HSP22 in cardiac overload pressure	HSP22 deletion accelerates transition to HF in the context of cardiac overload pressure
Rahsepar et al.^[^^72^^]^	To analyze the levels of HSP27 in patients with valvular heart disease	↑HSP27 in patients with valvular heart disease / ↑HSP27 may be useful as a biomarker in the assessment of HF
Raju et al.^[^^45^^]^	To investigate the regulation of HSP32 in the right-sided congestive heart failure model	Congestive heart failure ↑HSP32, but ↔HSP70
Rinaldi et al.^[^^8^^]^	To observe the effects of age and exercise on the antioxidant system and expression of HSP27 and HSP70	Physical exercise ↑HSP27 and ↑HSP70 / ↑HSP27 and ↑HSP70 ↓the deleterious effect of age on the antioxidant system of the heart
Rothenbacher et al.^[^^63^^]^	To investigate whether HSP60 is associated with heart disease	↑HSP60 does not seem to be an independent risk factor for coronary artery disease
Satoh et al.^[^^66^^]^	To determine the relationship between blood HSP70 and TLR4 after myocardial infarction	↑HSP70 in patients than controls / ↑HSP70 and ↑cytokines mediated TLR4 receptor
Schmitt et al.^[^^48^^]^	To investigate the synthesis of HSP70 in the human heart *in vivo* after CABG	↑HSP70 occurred after at least 2 hours of stress induction by CABG
Shinohara et al.^[^^26^^]^	To observe the effect of estrogen on the expression of HSP72 after ischemic damage	Estrogen ↓HSP72 induced by hyperthermia /↑HSP72 promotes ischemic protection
Staib et al.^[^^6^^]^	To investigate the effect of heat stress and mechanical overload on the HSP70 expression	Hyperthermia, regardless of workload, resulted in significant ↑HSP70 in the heart
Tanonaka et al.^[^^5^^]^	To evaluate the HSP72 production level during CHF and its relation to cardiac protection	Hyperthermia ↑HSP72 in the 2^nd^ week, but not in the 8^th^ week / ↑HSP72 was inversely correlated with worsening of the cardiac parameters / Advanced HF ↓HSP72
Tanonaka et al.^[^^43^^]^	To verify the expression of HSP during the development of HF	↑HSP27 and ↑HSP70 in the 1^st^ week, but not in the 8^th^ week / There was ↑HSP60 only in the 8^th^ week
Tanonaka et al.^[^^39^^]^	To examine the HSP72 expression in HF after acute myocardial infarction	The development of HF ↓HSP70 by hyperthermia
Ugurlucan et al.^[^^10^^]^	To investigate the effect of diabetes mellitus and glutamine administration on HSP70 expression in the heart	Diabetes ↑HSP70 in the myocardium / Parenteral administration of glutamine ↑HSP70
Veres et al.^[^^64^^]^	To explore the relationship between familial risk for heart disease and HSP60	↑HSP60 in the group with high familial risk / Children with ↑HSP60 showed higher values of *odds ratio* for heart disease / ↑HSP60 can be considered a relative risk factor for heart disease
Vittorini et al.^[^^14^^]^	To assess the HSP70 gene expression during blood cardioplegic arrest in children	Blood cardioplegia ↑HSP70 and protection of the ischemia/reperfusion injury
Wang et al.^[^^46^^]^	To investigate the regulation and transcription of HSP60 and HSP72 in HF	There were ↑HSP60 and hNF kB in HF / ↑HSP60 may be mediated by hNFkB / ↔HSP70 because ↔HSF1 in HF
Wei et al.^[^^15^^]^	To observe protein changes in hearts with HF and to identify potential biomarkers of this condition	↑HSP70 is the common feature of HF / ↑HSP70 may be used as a biomarker for the presence of HF and may hold diagnostic/prognostic potential in clinical practice
Wu et al.^[^^36^^]^	To compare HSP expression in patients with and without atrial fibrillation	↓HSP27 in patients with atrial fibrillation
Xi et al.^[^^37^^]^	To investigue whether the whole-body heat stress induces HSP70 expression and offers cardioprotection	Whole-body heat stress ↑HSP70 / No differences in HSP70 or control groups in infarct size after ischemia/reperfusion
Yamanaka et al.^[^^9^^]^	To investigate the effect of treatment with geranylgeranyl acetone (GGA) in the expression of HSP72	Treatment with GGA ↑HSP72 and ↑HSF-1 / ↑the recovery after ischemia/reperfusion / ↑HSP72 promoted cardioprotection
Yamashita et al.^[^^18^^]^	To compare the time course of tolerance to myocardial injury with the time course of HSP70 induction	↓infarct size in 48 and 72 hours after heat stress / ↑HSP70 content correlates with time course of cardioprotection
Yan et al.^[^^55^^]^	To investigate the expression of HSP60 in the heart, liver, and kidney of stressed broilers	↑HSP60 is specific and can be related to tissue damage in response to thermal stress / In the heart ↑HSP60 peaked at 5h after stress
Yu et al.^[^^54^^]^	To determine the HSP70 expression in cardiac and renal tissues of transport-stressed pigs	Transport stress ↑HSP70 in cardiac and renal tissues
Yu et al.^[^^50^^]^	To examine the expression of HSP70 after several periods of ischemia/reperfusion	Permanent ischemia and ischemia/reperfusion ↑HSP70 / ↑HSP70 from 30 min to 24h after ischemia/reperfusion
Zhang et al.^[^^70^^]^	To observe the correlation of HSP60 and CHD	↑HSP60 is related to the risk of CHD
Zhang et al.^[^^71^^]^	To determine whether HSP60, hypertension and diabetes have joint effects on CHD risk	↑HSP60 in the group with CHD than controls / ↑HSP60 is positively correlated with the risk and severity of CHD
Zhao et al.^[^^32^^]^	To examine the effects of probiotics-derived protein on heart injury	Probiotics-derived protein ↑HSP70 in heart cells and ↓ damage of ischemia/reperfusion
Zhong et al.^[^^11^^]^	To quantify the HSP70 levels hypoxia-induced and its relation to cardioprotection	Hypoxia ↑HSP70 and it provides protection against ischemia/reperfusion / ↑HSP70 can stay up to two weeks after hypoxia
Zhu and Wang^[^^29^^]^	To explore whether overexpression of HSP20 in cardiomyocytes protects against injury	↑HSP20 ↓the necrotic and ↓apoptotic cardiomyocytes

↑=increase; ↓=decrease; ↔ =no changes; CABG=coronary artery bypass grafting; CHD=coronary heart disease; CHF=chronic heart failure; CK=creatine kinase; HF=heart failure; ICH=intracerebral hemorrhage

## CONCLUSION

In summary, the literature provides consistent evidence that HSP20, HSP25, HSP27, HSP32 and HSP70 promote a protective effect following heart damage. Overexpression of these proteins in cardiac tissue increases protection as a natural result of ischemic damage, decreases infarcted area and myocardial apoptosis, and aids in heart recovery.

The increase in expression of these proteins can occur through some systemic stresses, such as hyper- and hypothermia, hypoxia, physical exercise and cardioplegia, as well as some substances and treatments, or the stress produced by heart disease. The sum of these findings could be useful under conditions in which it is necessary to induce ischemic damage, as in the case of surgery with cardiopulmonary bypass or other surgical procedures that include the temporary cutoff of supplies to the heart, in addition to improving cardiovascular endurance through heart disease. However, studies and procedures in human subjects still need to be more widely studied.

Although limited, knowledge on the role of HSPs as possible biomarkers has shown that HSP20, HSP27, HSP60 and HSP70 correlate well with heart disease, disease severity and resulting adverse events, the use of these proteins in the myocardium or in the blood is currently under evaluation as predictive markers in pre- and post-surgery. Little or no reference has been found in the literature, however, about the possibility of manipulating the production of these repair proteins. However, a likely practical application for the use of HSPs could be available if we could either enhance the overexpression by specific diet or avoid the use of practices or therapeutic procedures that could jeopardize the expression of HSPs and their benefits. While our understanding of these major HSPs in heart disease is incomplete, there is a clear potential role for the therapeutic modulation of HSPs in the practical clinical context. In the absence of such data, further studies would be required to better explore this natural repair system, perhaps even as a tool to evaluate the success of the therapy.

**Table:** 

Authors' roles & responsibilities	
VASJ	Substantial contributions to the conception or design of the work; or the acquisition, analysis, or interpretation of data for the work; drafting the work or revising it critically for important intellectual content; final approval of the version to be published
PCBL	Substantial contributions to the conception or design of the work; or the acquisition, analysis, or interpretation of data for the work; drafting the work or revising it critically for important intellectual content; final approval of the version to be published
MAC	Substantial contributions to the conception or design of the work; or the acquisition, analysis, or interpretation of data for the work; drafting the work or revising it critically for important intellectual content; final approval of the version to be published
CSM	Substantial contributions to the conception or design of the work; or the acquisition, analysis, or interpretation of data for the work; drafting the work or revising it critically for important intellectual content; final approval of the version to be published
JAF	Substantial contributions to the conception or design of the work; or the acquisition, analysis, or interpretation of data for the work; drafting the work or revising it critically for important intellectual content; final approval of the version to be published
PNM	Substantial contributions to the conception or design of the work; or the acquisition, analysis, or interpretation of data for the work; drafting the work or revising it critically for important intellectual content; final approval of the version to be published

## References

[1] Kampinga HH, Hageman J, Vos MJ, Kubota H, Tanquay RM, Bruford EA (2009). Guidelines for the nomenclature of the human heat shock
proteins. Cell Stress Chaperones.

[2] Jolly C, Morimoto RI (2000). Role of the heat shock response and molecular chaperones in
oncogenesis and cell death. J Natl Cancer Inst.

[3] Jiang BH, Jiang G, Zheng JZ, Lu Z, Hunter T, Vogt PK (2001). Phosphatidylinositol 3-kinase signaling controls levels of
hypoxia-inducible factor 1. Cell Growth Differ.

[4] Beere HM (2005). Death versus survival: functional interaction between the
apoptotic and stress-inducible heat shock protein pathways. J Clin Invest.

[5] Tanonaka K, Furuhama KI, Yoshida H, Kakuta K, Miyamoto Y, Toga W (2001). Protective effect of heat shock protein 72 on contractile
function of perfused failing heart. Am J Physiol Heart Circ Physiol.

[6] Staib JL, Quindry JC, French JP, Criswell DS, Powers SK (2007). Increased temperature, not cardiac load, activates heat shock
transcription factor 1 and heat shock protein 72 expression in the
heart. Am J Physiol Regul Integr Comp Physiol.

[7] Marunouchi T, Murata M, Takagi N, Tanonaka K (2013). Possible involvement of phosphorylated heat-shock factor-1 in
changes in heat shock protein 72 induction in the failing rat heart
following myocardial infarction. Biol Pharm Bull.

[8] Rinaldi B, Corbi G, Boccuti S, Filippelli W, Rengo G, Leosco D (2006). Exercise training affects age-induced changes in SOD and heat
shock protein expression in rat heart. Exp Gerontol.

[9] Yamanaka K, Takahashi N, Ooie T, Kaneda K, Yoshimatsu H, Saikawa T (2003). Role of protein kinase C in geranylgeranylacetone-induced
expression of heat-shock protein 72 and cardioprotection in the rat
heart. J Mol Cell Cardiol.

[10] Ugurlucan M, Erer D, Karatepe O, Ziyade S, Haholu A, Gungor Ugurlucan F (2010). Glutamine enhances the heat shock protein 70 expression as a
cardioprotective mechanism in left heart tissues in the presence of diabetes
mellitus. Expert Opin Ther Targets.

[11] Zhong N, Zhang Y, Fang QZ, Zhou ZN (200). Intermittent hypoxia exposure-induced heat-shock protein 70
expression increases resistance of rat heart to ischemic
injury. Acta Pharmacol Sin.

[12] Okubo S, Wildner O, Shah MR, Chelliah JC, Hess ML, Kukreja RC (2001). Gene transfer of heat-shock protein 70 reduces infarct size in
vivo after ischemia/reperfusion in the rabbit heart. Circulation.

[13] Kwon JH, Kim JB, Lee KH, Kang SM, Chung N, Jang Y (2007). Protective effect of heat shock protein 27 using protein
transduction domain-mediated delivery on ischemia/reperfusion heart
injury. Biochem Biophys Res Commun.

[14] Vittorini S, Storti S, Andreani G, Giusti L, Murzi B, Furfori P (2007). Heat shock protein 70-1 gene expression in pediatric heart
surgery using blood cardioplegia. Clin Chem Lab Med.

[15] Wei YJ, Huang YX, Shen Y, Cui CJ, Zhang XL, Zhang H (2009). Proteomic analysis reveals significant elevation of heat shock
protein 70 in patients with chronic heart failure due to arrhythmogenic
right ventricular cardiomyopathy. Mol Cell Biochem.

[16] Bonanad C, Núñez J, Sanchis J, Bodi V, Chaustre F, Chillet M (2013). Serum heat shock protein 60 in acute heart failure: a new
biomarker?. Congest Heart Fail.

[17] Li Z, Song Y, Xing R, Yu H, Zhang Y, Li Z (2013). Heat shock protein 70 acts as a potential biomarker for early
diagnosis of heart failure. PLoS One.

[18] Yamashita N, Hoshida S, Nishida M, Igarashi J, Aoki K, Hori M (1997). Time course of tolerance to ischemia-reperfusion injury and
induction of heat shock protein 72 by heat stress in the rat
heart. J Mol Cell Cardiol.

[19] Kukreja RC, Qian YZ, Okubo S, Flaherty EE (1999). Role of protein kinase C and 72 kDa heat shock protein in
ischemic tolerance following heat stress in the rat heart. Mol Cell Biochem.

[20] Qian YZ, Bernardo NL, Nayeem MA, Chelliah J, Kukreja RC (1999). Induction of 72-kDa heat shock protein does not produce second
window of ischemic preconditioning in rat heart. Am J Physiol.

[21] Meldrum DR, Meng X, Shames BD, Pomerantz B, Donnahoo KK, Banerjee A (1999). Liposomal delivery of heat-shock protein 72 into the heart
prevents endotoxin-induced myocardial contractile
dysfunction. Surgery.

[22] Nomura F, Aoki M, Forbess JM, Mayer Jr JE (1999). Myocardial self-preservative effect of heat shock protein 70 on
an immature lamb heart. Ann Thorac Surg.

[23] Kim SO, Baines CP, Critz SD, Pelech SL, Katz S, Downey JM (1999). Ischemia induced activation of heat shock protein 27 kinases and
casein kinase 2 in the preconditioned rabbit heart. Biochem Cell Biol.

[24] Ooie T, Takahashi N, Saikawa T, Nawata T, Arikawa M, Yamanaka K (2001). Single oral dose of geranylgeranylacetone induces heat-shock
protein 72 and renders protection against ischemia/reperfusion injury in rat
heart. Circulation.

[25] Efthymiou CA, Mocanu MM, Belleroche J, Wells DJ, Latchmann DS, Yellon DM (2004). Heat shock protein 27 protects the heart against myocardial
infarction. Basic Res Cardiol.

[26] Shinohara T, Takahashi N, Ooie T, Ichinose M, Hara M, Yonemochi H (2004). Estrogen inhibits hyperthermia-induced expression of heat-shock
protein 72 and cardioprotection against ischemia/reperfusion injury in
female rat heart. J Mol Cell Cardiol.

[27] Chen H, Wu XJ, Lu XY, Zhu L, Wang LP, Yang HT (2005). Phosphorylated heat shock protein 27 is involved in enhanced
heart tolerance to ischemia in short-term type 1 diabetic
rats. Acta Pharmacol Sin.

[28] Gauthaman K, Banerjee SK, Dinda AK, Ghosh CC, Maulik SK (2005). Terminalia arjuna (Roxb.) protects rabbit heart against
ischemic-reperfusion injury: role of antioxidant enzymes and heat shock
protein. J Ethnopharmacol.

[29] Zhu YH, Wang X (2005). Overexpression of heat-shock protein 20 in rat heart myogenic
cells confers protection against simulated ischemia/reperfusion
injury. Acta Pharmacol Sin.

[30] Kohno H, Takahashi N, Shinohara T, Ooie T, Yufu K, Nakagawa M (2007). Receptor-mediated suppression of cardiac heat-shock protein 72
expression by testosterone in male rat heart. Endocrinology.

[31] Krishnamurthy K, Kanagasabai R, Druhan LJ, Ilangovan G (2012). Heat shock protein 25-enriched plasma transfusion preconditions
the heart against doxorubicin-induced dilated cardiomyopathy in
mice. J Pharmacol Exp Ther.

[32] Zhao B, Sun G, Feng G, Duan W, Zhu X, Chen S (2012). Carboxy terminus of heat shock protein (HSP) 70-interacting
protein (CHIP) inhibits HSP70 in the heart. J Physiol Biochem.

[33] Jiang B, Zhang B, Liang P, Chen G, Zhou B, Lv C (2013). Nucleolin protects the heart from ischaemia-reperfusion injury by
up-regulating heat shock protein 32. Cardiovasc Res.

[34] Li Q, Shi M, Li B (2013). Anandamide enhances expression of heat shock protein 72 to
protect against ischemia-reperfusion injury in rat heart. J Physiol Sci.

[35] McGinley LM, McMahon J, Stocca A, Duffy A, Flynn A, O'Toole D (2013). Mesenchymal stem cell survival in the infarcted heart is enhanced
by lentivirus vector-mediated heat shock protein 27
expression. Hum Gene Ther.

[36] Wu W, Lu Z, Li Y, Chen Z, Jiang H, Li Y (2015). Decreased Cardiac Expression of Heat Shock Protein 27 is
Associated with Atrial Fibrillation in Patients with Rheumatic Heart
Disease. Acta Cardiol Sin.

[37] Xi L, Chelliah J, Nayeem MA, Levasseur JE, Hess ML, Kukreja RC (1998). Whole body heat shock fails to protect mouse heart against
ischemia/reperfusion injury: role of 72 kDa heat shock protein and
antioxidant enzymes. J Mol Cell Cardiol.

[38] Moalic JM, Bauters C, Himbert D, Bercovici J, Mouas C, Guicheney P (1989). Phenylephrine, vasopressin and angiotensin II as determinants of
proto-oncogene and heat-shock protein gene expression in adult rat heart and
aorta. J Hypertens.

[39] Tanonaka K, Toga W, Takahashi M, Yoshida H, Oikawa R, Takeo S (2004). Induction of heat shock protein 72 in the failing heart is
attenuated after an exposure to heat shock. Mol Cell Biochem.

[40] Kukreja RC, Kontos MC, Loesser KE, Batra SK, Qian YZ, Gbur Jr CJ (1994). Oxidant stress increases heat shock protein 70 mRNA in isolated
perfused rat heart. Am J Physiol.

[41] Ferrari R, Bongrazio M, Cargnoni A, Comini L, Pasini E, Gaia G (1996). Heat shock protein changes in hibernation: a similarity with
heart failure?. J Mol Cell Cardiol.

[42] Comini L, Gaia G, Curello S, Ceconi C, Pasini E, Benigno M (1996). Right heart failure chronically stimulates heat shock protein 72
in heart and liver but not in other tissues. Cardiovasc Res.

[43] Tanonaka K, Toga W, Yoshida H, Takeo S (2003). Myocardial heat shock protein changes in the failing heart
following coronary artery ligation. Heart Lung Circ.

[44] Osaki J, Haneda T, Kashiwagi Y, Oi S, Fukuzawa J, Sakai H (1998). Pressure-induced expression of heat shock protein 70 mRNA in
adult rat heart is coupled both to protein kinase A-dependent and protein
kinase C-dependent systems. J Hypertens.

[45] Raju VS, Imai N, Liang CS (1999). Chamber-specific regulation of heme oxygenase-1 (heat shock
protein 32) in right-sided congestive heart failure. J Mol Cell Cardiol.

[46] Wang Y, Chen L, Hagiwara N, Knowlton AA (2010). Regulation of heat shock protein 60 and 72 expression in the
failing heart. J Mol Cell Cardiol.

[47] Knowlton AA, Sun L (2001). Heat-shock factor-1, steroid hormones, and regulation of
heat-shock protein expression in the heart. Am J Physiol Heart Circ Physiol.

[48] Schmitt JP, Schunkert H, Birnbaum DE, Aebert H (2002). Kinetics of heat shock protein 70 synthesis in the human heart
after cold cardioplegic arrest. Eur J Cardiothorac Surg.

[49] Dybdahl B, Wahba A, Lien E, Flo TH, Waage A, Qureshi N (2002). Inflammatory response after open heart surgery: release of
heat-shock protein 70 and signaling through toll-like
receptor-4. Circulation.

[50] Yu H, Yokoyama M, Asano G (1999). Time course of expression and localization of heat shock protein
72 in the ischemic and reperfused rat heart. Jpn Circ J.

[51] Gray CC, Amrani M, Smolenski RT, Nakamura K, Yacoub MH (2001). Cold cardioplegic arrest enhances heat shock protein 70 in the
heat-shocked rat heart. J Thorac Cardiovasc Surg.

[52] Katayose D, Isoyama S, Fujita H, Shibahara S (1993). Separate regulation of heme oxygenase and heat shock protein 70
mRNA expression in the rat heart by hemodynamic stress. Biochem Biophys Res Commun.

[53] Knowlton AA, Eberli FR, Brecher P, Romo GM, Owen A, Apstein CS (1991). A single myocardial stretch or decreased systolic fiber
shortening stimulates the expression of heat shock protein 70 in the
isolated, erythrocyte-perfused rabbit heart. J Clin Invest.

[54] Yu H, Bao ED, Zhao RQ, Lv QX (2007). Effect of transportation stress on heat shock protein 70
concentration and mRNA expression in heart and kidney tissues and serum
enzyme activities and hormone concentrations of pigs. Am J Vet Res.

[55] Yan J, Bao E, Yu J (2009). Heat shock protein 60 expression in heart, liver and kidney of
broilers exposed to high temperature. Res Vet Sci.

[56] Hoppichler F, Lechleitner M, Traweger C, Schett G, Dzien A, Sturm W (1996). Changes of serum antibodies to heat-shock protein 65 in coronary
heart disease and acute myocardial infarction. Atherosclerosis.

[57] Dohke T, Wada A, Isono T, Fujii M, Yamamoto T, Tsutamoto T (2006). Proteomic analysis reveals significant alternations of cardiac
small heat shock protein expression in congestive heart
failure. J Card Fail.

[58] Hu H, Wang L, Okauchi M, Keep RF, Xi G, Hua Y (2011). Deferoxamine affects heat shock protein expression in heart after
intracerebral hemorrhage in aged rats. Acta Neurochir Suppl.

[59] Boluyt MO, Brevick JL, Rogers DS, Randall MJ, Scalia AF, Li ZB (2006). Changes in the rat heart proteome induced by exercise training:
Increased abundance of heat shock protein hsp20. Proteomics.

[60] Almela P, Martínez-Laorden E, Atucha NM, Milanés MV, Laorden ML (2011). Naloxone-precipitated morphine withdrawal evokes phosphorylation
of heat shock protein 27 in rat heart through extracellular signal-regulated
kinase. J Mol Cell Cardiol.

[61] Latif N, Yacoub MH, Dunn MJ (1997). Association of pretransplant anti-heart antibodies against human
heat shock protein 60 with clinical course following cardiac
transplantation. Transplant Proc.

[62] Baba HA, Schmid KW, Schmid C, Blasius S, Heinecke A, Kerber S (1998). Possible relationship between heat shock protein 70, cardiac
hemodynamics, and survival in the early period after heart
transplantation. Transplantation.

[63] Rothenbacher D, Hoffmeister A, Bode G, Miller M, Koenig W, Brenner H (2001). Helicobacter pylori heat shock protein 60 and risk of coronary
heart disease: a case control study with focus on markers of systemic
inflammation and lipids. Atherosclerosis.

[64] Veres A, Szamosi T, Ablonczy M, Szamosi Jr T, Singh M, Karádi I (2002). Complement activating antibodies against the human 60 kDa heat
shock protein as a new independent family risk factor of coronary heart
disease. Eur J Clin Invest.

[65] Genth-Zotz S, Bolger AP, Kalra PR, von Haehling S, Doehner W, Coats AJ (2004). Heat shock protein 70 in patients with chronic heart failure:
relation to disease severity and survival. Int J Cardiol.

[66] Satoh M, Shimoda Y, Akatsu T, Ishikawa Y, Minami Y, Nakamura M (2006). Elevated circulating levels of heat shock protein 70 are related
to systemic inflammatory reaction through monocyte Toll signal in patients
with heart failure after acute myocardial infarction. Eur J Heart Fail.

[67] Gombos T, Förhécz Z, Pozsonyi Z, Jánoskuti L, Prohászka Z (2008). Interaction of serum 70-kDa heat shock protein levels and HspA1B
(+1267) gene polymorphism with disease severity in patients with chronic
heart failure. Cell Stress Chaperones.

[68] Jafarzadeh A, Esmaeeli-Nadimi A, Shariati M (2008). High sensitivity C-reactive protein and immunoglobulin G against
Chlamydia pneumoniae and chlamydial heat shock protein-60 in ischemic heart
disease. Iran J Immunol.

[69] Niizeki T, Takeishi Y, Watanabe T, Nitobe J, Miyashita T, Miyamoto T (2008). Relation of serum heat shock protein 60 level to severity and
prognosis in chronic heart failure secondary to ischemic or idiopathic
dilated cardiomyopathy. Am J Cardiol.

[70] Zhang X, He M, Cheng L, Chen Y, Zhou L, Zeng H (2008). Elevated heat shock protein 60 levels are associated with higher
risk of coronary heart disease in Chinese. Circulation.

[71] Zhang X, He MA, Cheng L, Zhou L, Zeng H, Wang J (2008). Joint effects of antibody to heat shock protein 60, hypertension,
and diabetes on risk of coronary heart disease in Chinese. Clin Chem.

[72] Rahsepar AA, Mirzaee A, Moodi F, Moohebati M, Tavallaie S, Eshraghi A (2012). Anti-heat shock protein 27 titers and oxidative stress levels are
elevated in patients with valvular heart disease. Angiology.

[73] Qiu H, Lizano P, Laure L, Sui X, Rashed E, Park JY (2011). H11 kinase/heat shock protein 22 deletion impairs both nuclear
and mitochondrial functions of STAT3 and accelerates the transition into
heart failure on cardiac overload. Circulation.

